# Genome-Wide Characterization of the Apple HD-Zip IV Gene Family and Functional Validation of MdHDZIV3 Under PEG-Induced Osmotic Stress

**DOI:** 10.3390/plants15172670

**Published:** 2026-08-31

**Authors:** An Yang, Xuqing Tong, Songya Ma, Chenglong Liu, Bichen Wang, Lin Guo, Hui Li, Zitong He, Liqun Kang, Xiaolei Han, Caixia Zhang

**Affiliations:** 1 Key Laboratory of Horticulture Crops Germplasm Resources Utilization, Research Institute of Pomology, Chinese Academy of Agricultural Sciences (CAAS), Xingcheng 125100, China; yangan19950602@163.com (A.Y.); m13176909620@163.com (X.T.); masongya@caas.cn (S.M.); long17866711903@163.com (C.L.); w2787414313@foxmail.com (B.W.); 821012450315@caas.cn (L.G.); 15866715612@163.com (H.L.); hzt1059540772@163.com (Z.H.); kangliqun@caas.cn (L.K.); 2 Key Laboratory of Horticultural Crops Germplasm Resources Utilization, Ministry of Agriculture and Rural Affairs of the People’s Republic of China, Xingcheng 125100, China

**Keywords:** homeodomain–leucine zipper IV, transcription factor, genome-wide analysis, abiotic stress

## Abstract

The homeodomain–leucine zipper IV (HD-Zip IV) transcription factor subfamily plays essential roles in epidermal development, cuticle formation, lipid metabolism, and environmental adaptation in plants. Despite its biological importance, the HD-Zip IV family has not been systematically characterized in apple (*Malus domestica*). Here, we identified 17 apple HD-Zip IV genes and named them *MdHDZIV1*–*MdHDZIV17* based on their locations on the chromosomes. The 17 genes showed a nonuniform distribution on eight chromosomes, while the occurrence of both tandem and segmental duplications indicated that family expansion involved more than one duplication mechanism. All MdHDZIV proteins contained the conserved HD, LZ, START, and SAD domains but lacked the MEKHLA domain, consistent with typical HD-Zip IV structural features. Phylogenetic analysis classified MdHDZIV proteins into five groups together with HD-Zip IV members from Arabidopsis thaliana and rice, indicating evolutionary conservation of this subfamily. Collinearity and Ka/Ks analyses revealed that duplicated *MdHDZIV* gene pairs were mainly subjected to purifying selection. Promoter scanning revealed diverse cis-regulatory motifs associated with hormonal signaling, environmental stress, light response, and epidermal regulation, including ABRE, ARE, W-box, MYC, G-box, and L1-box motifs. Integration of transcriptomic profiling with qRT-PCR validation revealed pronounced tissue-dependent differences in the expression of *MdHDZIV* genes in leaf, fruit skin, and branch bark. Under PEG_6000_-induced osmotic stress and NaCl-induced salt stress, 10 candidate *MdHDZIV* genes displayed gene-specific and stress type-specific expression patterns, with *MdHDZIV3* showing strong induction under PEG_6000_ treatment. Functional validation in apple calli showed that *MdHDZIV3* overexpression enhanced PEG tolerance, increased fresh weight, elevated SOD and POD activities, and reduced MDA accumulation under osmotic stress. These findings provide a genome-wide framework for understanding the apple HD-Zip IV gene family.

## 1. Introduction

The plant epidermis is the outermost tissue directly exposed to the external environment and plays essential roles in maintaining organ structure, limiting water loss, defending against pathogen invasion, and enabling adaptation to environmental stresses [1,2]. The plant surface is protected by a cuticular layer consisting primarily of cutin and waxes, whose hydrophobic properties help maintain barrier function and improve adaptation to environmental conditions [3,4]. Cuticular wax production and deposition are controlled by organ developmental processes, environmental signals, and transcriptional regulatory networks, and exhibit pronounced tissue specificity across external organs such as leaf, stem bark, and fruit surfaces [5,6,7,8,9]. Given these roles, identifying key regulatory factors involved in epidermal development, wax biosynthesis, and stress responses is crucial for elucidating the mechanisms underlying epidermal barrier formation and plant adaptation to environmental conditions.

Transcription factors (TFs) act upstream of numerous pathways controlling epidermal differentiation and cuticular wax biosynthesis. Within this broader group, HD-Zip proteins are plant-specific TFs with extensive functions in growth, organ formation, developmental control, and responses to environmental signals [10,11]. Two conserved modules define HD-Zip proteins: the HD region mediates sequence-selective DNA binding, whereas the LZ region promotes dimerization and influences transcription of target genes [12]. On the basis of structural traits and phylogenetic relationships, the family is separated into four subfamilies, HD-Zip I–IV [13]. In addition to the canonical HD and LZ domains, HD-Zip IV proteins contain C-terminal START and SAD domains that distinguish them from other HD-Zip groups. In contrast to proteins belonging to the HD-Zip III group, HD-Zip IV members do not possess a MEKHLA domain, providing an important structural criterion for separating the two groups [12,14]. The START-SAD region may enable HD-Zip IV proteins to participate in lipid perception, signaling, and developmental processes associated with the epidermis [15]. Consistent with this structural feature, previous studies have shown that HD-Zip IV genes are preferentially transcribed in epidermal and subepidermal layers and function in epidermal cell fate determination, cuticle formation, wax metabolism, and environmental responses [15,16,17]. Together, these properties position HD-Zip IV proteins as regulatory links among epidermal development, lipid-related metabolism, and stress acclimation.

Functional studies across diverse plant species have further demonstrated the relatively conserved roles of the HD-Zip IV proteins in epidermis-related developmental processes. In *Arabidopsis thaliana*, *ATML1* and *PDF2* regulate epidermal cell specification and tissue maintenance and perform partially overlapping functions during shoot epidermis differentiation [18,19]. Notably, *ATML1* not only participates in epidermal cell fate determination but also shows dynamic expression patterns closely associated with the formation and maintenance of specific epidermal cell types, underscoring the importance of HD-Zip IV factors in epidermal patterning [15,20]. Comparable activities of HD-Zip IV proteins have been reported in plant species beyond Arabidopsis. In maize, most HD-Zip IV genes are highly transcribed in epidermal tissues and control genes associated with lipids and cuticle formation, consistent with a role in building the epidermal barrier [21]. In rice, members such as *ROC5*, *ROC8*, and *ROC1* are closely associated with leaf epidermal cell development, bulliform cell size regulation, leaf rolling, and drought response, indicating that HD-Zip IV proteins contribute not only to organ morphogenesis but also to adaptation to abiotic stresses [17,22,23]. In cotton, HD-Zip IV members including *GaHOX1* and *GhHOX3* are involved in fiber initiation and elongation, further demonstrating their roles in the formation of specialized epidermal cells [24,25]. Additionally, studies in tomato and other crop species suggest that HD-Zip IV-related factors participate in fruit epidermal structure formation, cuticle development, and the regulation of epidermal barrier function [26]. Overall, although HD-Zip IV members exhibit species-specific regulatory targets and mechanisms, their functions are repeatedly linked to epidermal cell specification, cuticle development, lipid metabolism, and environmental responses. Taken together, these observations establish the HD-Zip IV subfamily as a major class of transcriptional regulators that integrates epidermal development with environmental acclimation.

Given these conserved functions, HD-Zip IV genes are likely to contribute to epidermal barrier establishment and wax metabolism in perennial fruit crops such as apple. As an important fruit crop, apple develops specialized epidermal barriers in leaves, branch bark, and fruit skin, and wax deposition in these tissues contributes to water conservation, surface defense, and environmental acclimation. Earlier studies have linked variation in apple wax composition, abundance, and deposition to developmental stage, epidermal architecture, and environmental conditions [27,28,29]. Progress in research on apple epidermal biology and wax metabolism has led to the discovery of a growing set of regulators governing cuticle formation, wax biosynthesis, and stress responses. For instance, *MdSHN3* has been shown to promote cuticle formation in apple fruit and influence epidermal integrity [30]; MdMYB30 directly regulates *MdKCS1* and participates in surface wax biosynthesis [31]; and MdSIZ1 enhances *MdKCS1* expression and wax accumulation by stabilizing MdMYB30 through SUMOylation [32]. Natural sequence variation in *MdHDG5* has recently been linked to differences in apple leaf cuticular-wax deposition, providing genetic support for participation of HD-Zip IV TFs in epidermal wax regulation [16]. Despite these advances, systematic studies on the HD-Zip IV subfamily in apple remain limited. Information remains incomplete regarding family composition, protein architecture, evolutionary history, organ-dependent expression, and possible functions in epidermal development, wax deposition, and environmental adaptation.

We therefore aimed to identify HD-Zip IV genes across the apple genome and characterize their physicochemical attributes, domain architecture, exon–intron structures, conserved motifs, chromosomal distribution, synteny, and promoter cis-elements. Furthermore, public transcriptomic datasets were used to examine organ-dependent expression of these genes in roots, leaves, flowers, fruits, and seeds. Selected genes were further evaluated by qRT-PCR in fruit skin and branch bark, two tissues associated with epidermal barrier formation. In addition, apple seedlings were subjected to PEG_6000_ and NaCl treatments to evaluate the transcriptional responses of selected HD-Zip IV genes under different environmental conditions. Together, this study aims to provide a comprehensive understanding of the genomic characteristics, expression patterns, and potential biological functions of the apple HD-Zip IV family, thereby providing a foundation for future studies on epidermal development, wax metabolism and stress adaptation.

## 2. Results

### 2.1. Identification and Characterization of HD-ZIP IV Family Genes in Apple

**Figure 1:**
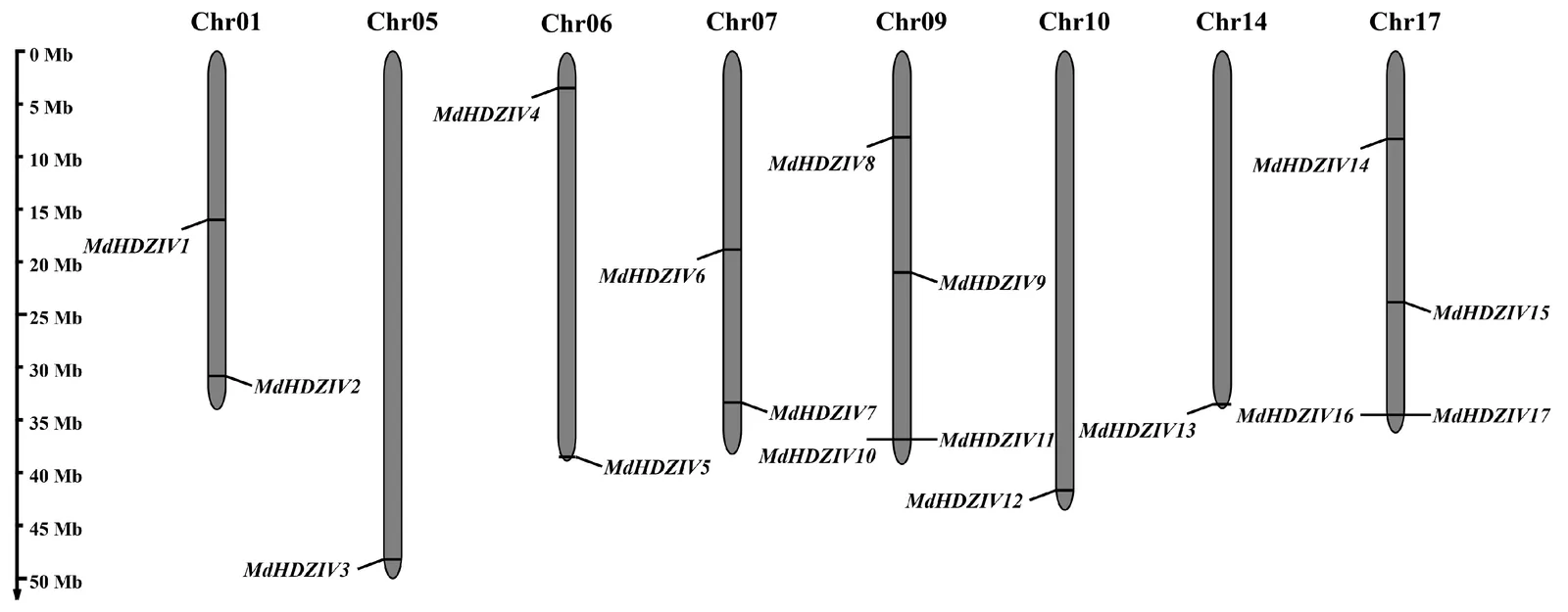
Chromosomal localization of MdHDZIVs. The chromosome map was drawn by MG2C.

Genome-wide identification of HD-ZIP IV family members in apple (*Malus domestica*) was performed using a conserved-domain-based approach. The HMM models corresponding to the HD domain (PF00046) and START domain (PF01852) were obtained from the Pfam database and applied to search the GDDH13_v1.1 protein dataset. Candidate proteins were further examined using the NCBI CD-Search tool to verify the presence and integrity of conserved domains (Table S1). Proteins containing the MEKHLA domain (PF08670), a characteristic feature of HD-ZIP III members, were excluded. A total of 17 HD-ZIP IV genes were ultimately identified in the apple genome and renamed *MdHDZIV1* to *MdHDZIV17* according to their chromosomal positions (Figure 1). Mapping of *MdHDZIV* genes onto the apple genome revealed their preferential distribution across eight chromosomes, with chromosome 9 carrying the largest number of family members. Tandem duplication events were detected on chromosomes 9 and 17, indicating that local duplication likely participated in the expansion of this family. The physicochemical properties of the MdHDZIV proteins were further analyzed using EMBOSS (version 6.6.0) (Table 1). The predicted MdHDZIV proteins ranged from 639 to 834 amino acids in length, with molecular weights varying from 71.66 to 92.82 kDa. Among them, MdHDZIV4 and MdHDZIV17 encoded the largest and smallest proteins, respectively. Predicted pI values ranged from 5.19 for MdHDZIV14 to 7.37 for MdHDZIV16; values below 7 for most family members indicate a predominantly acidic character. In addition, GRAVY scores ranged between −0.497 (MdHDZIV9) and −0.195 (MdHDZIV7), classifying every MdHDZIV protein as hydrophilic.

### 2.2. Conserved Domain and Phylogenetic Analyses of MdHDZIVs

**Figure 2:**
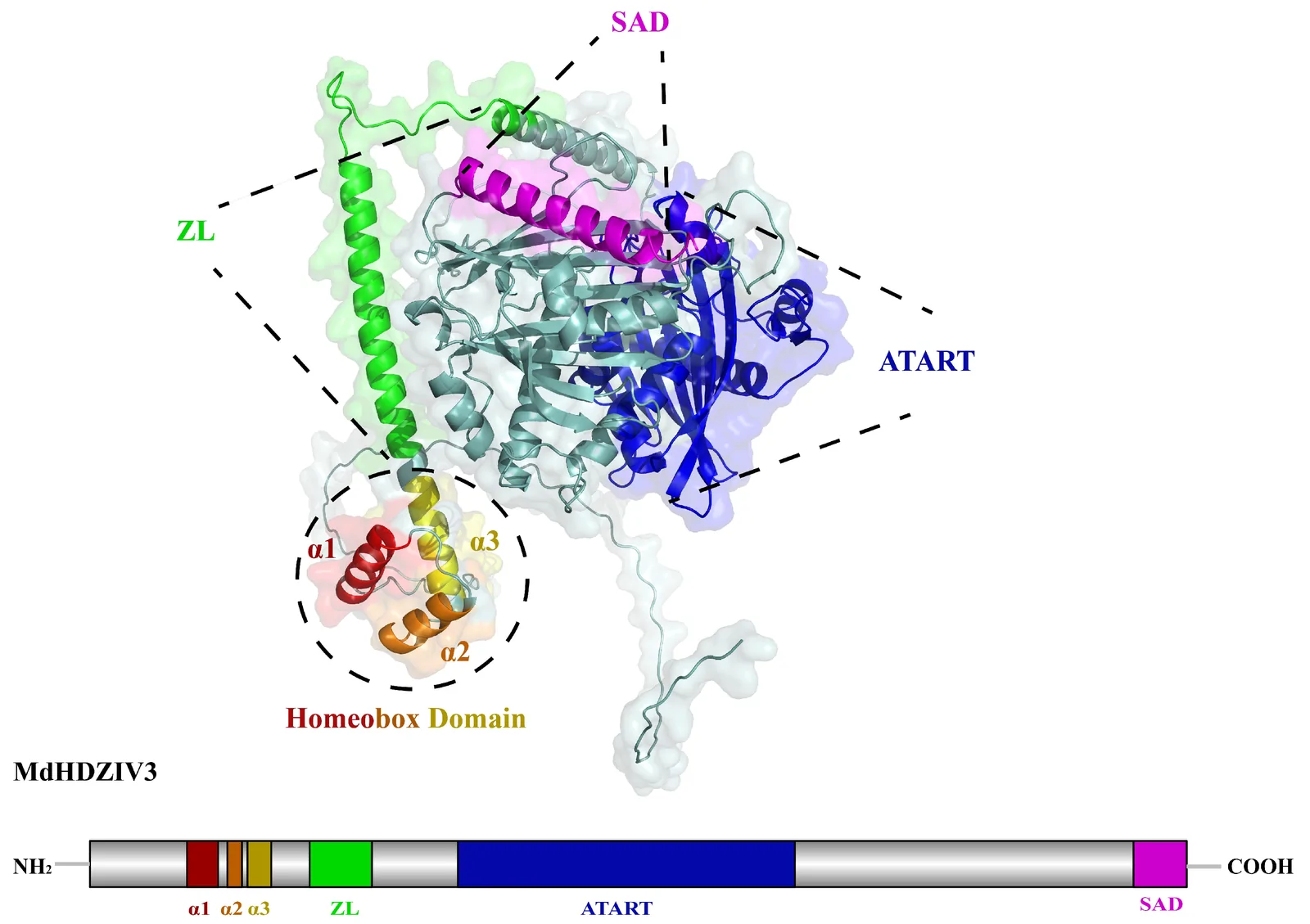
Protein structure diagram of *MdHDZIVs*. Genes and 3D structure representation of MdHD-Zip IV proteins. The conserved Homeobox Domain consists of three α-helices (α1–α3), colored red, orange, and yellow, respectively. The ZL region, ATART motif, and START-associated domain (SAD) are rep-resented by green, blue, and purple colors, respectively.

Using MdHDZIV3 as a representative protein, three-dimensional structure prediction with AlphaFold3 revealed the conserved modular architecture characteristic of HD-ZIP IV proteins (Figure 2). The Homeobox domain located at the N terminus is formed by three α-helical segments (α1–α3) and is mainly involved in sequence-specific DNA recognition. This region is followed by a continuous α-helical leucine zipper (LZ) domain. Together, the Homeobox and LZ domains are closely arranged and form a compact N-terminal module. In contrast, the C-terminal region contains the START and SAD domains, which together form a compact globular conformation that may participate in ligand recognition and conformational stabilization. Overall, this predicted structural organization is highly consistent with that of typical HD-ZIP IV proteins, such as PDF2, ATML1, and GL2 in *Arabidopsis thaliana*.

**Figure 3:**
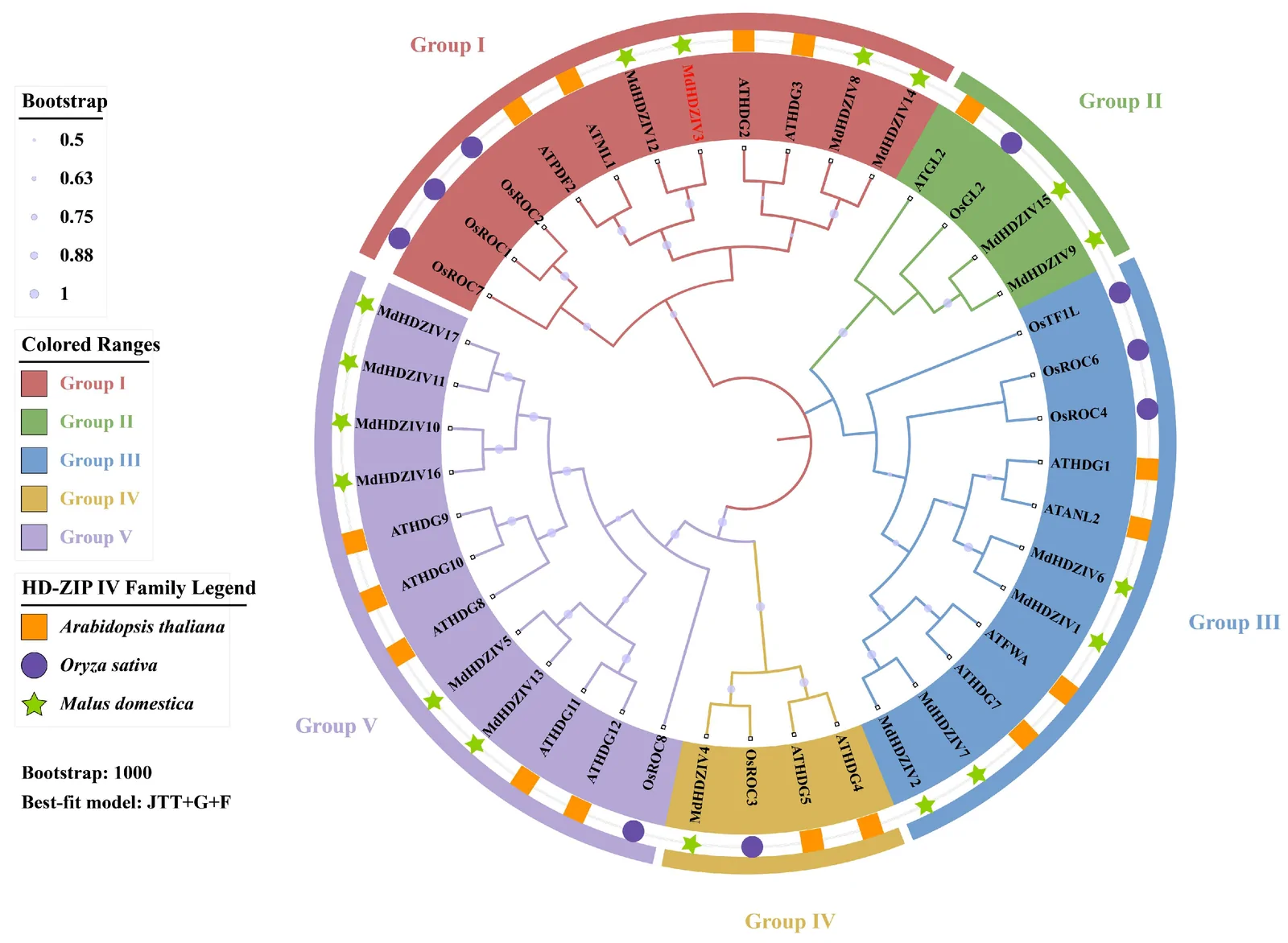
Phylogenetic analysis of the HD-ZIP IV proteins. An ML tree was inferred from complete MdHDZIV amino acid sequences and evaluated with 1000 bootstrap iterations. Phylogenetic analysis resolved the 17 MdHDZIV proteins into five distinct clades (Groups I–V).

To examine evolutionary divergence among the 17 apple proteins, full-length HD-Zip IV sequences from *Arabidopsis* and rice were combined with the *MdHDZIV* sequences to generate an ML phylogeny tree (Figure 3). Tree topology and bootstrap support separated the HD-Zip IV proteins into five clades (Groups I–V). All 17 apple members occurred across the five clades, consistent with strong conservation of HD-Zip IV proteins over the course of plant evolution. Among the five groups, Group V contained the largest number of MdHDZIV members, including MdHDZIV5, MdHDZIV10, MdHDZIV11, MdHDZIV13, MdHDZIV16, and MdHDZIV17, followed by Group I (MdHDZIV3, MdHDZIV8, MdHDZIV12, and MdHDZIV14) and Group III (MdHDZIV1, MdHDZIV2, MdHDZIV6, and MdHDZIV7). In contrast, Group II contained only two members, MdHDZIV9 and MdHDZIV15, whereas Group IV contained only one member, MdHDZIV4. Notably, MdHDZIV3 clustered closely with the *Arabidopsis* proteins PDF2 and ATML1, implying a close evolutionary relationship and potentially related biological activities.

**Figure 4:**
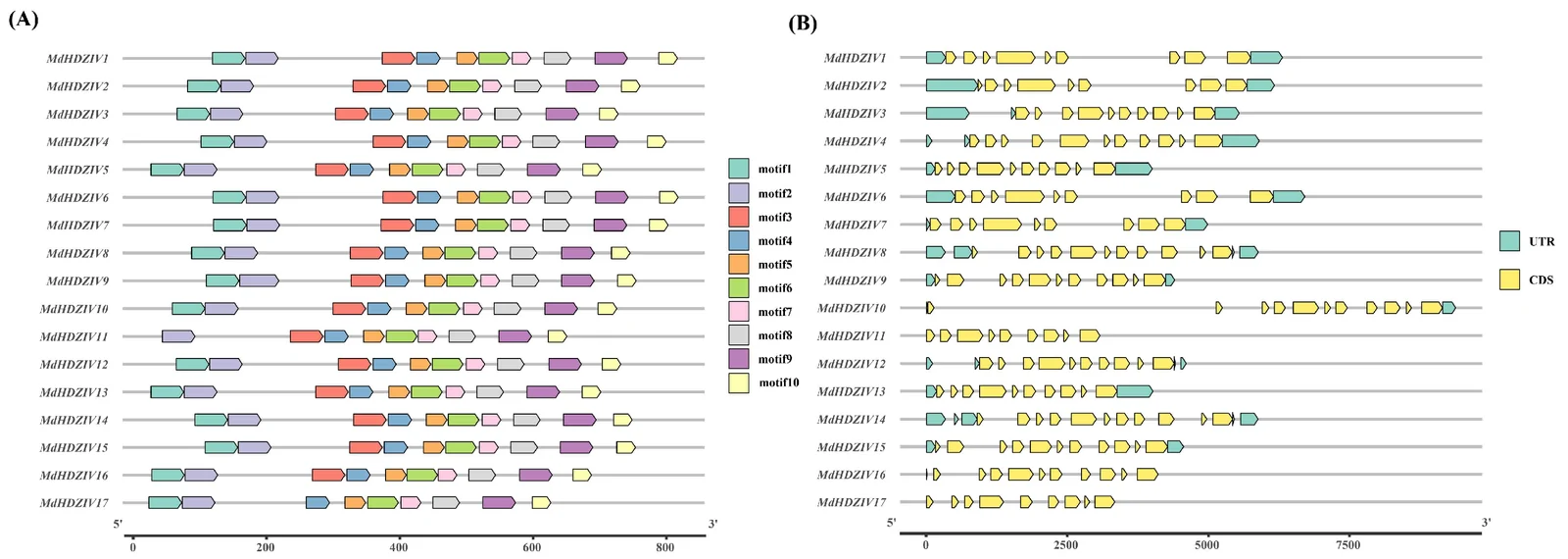
Exon–intron organization and conserved motifs of *MdHDZIV* genes and proteins. (**A**) Conserved motif analysis of HDZIVs in apples. The left panel presents the MEME-derived motifs, with individual colors representing different motif types. (**B**) Exon–intron organization of the apple HD-ZIP IV genes. Yellow rectangles denote CDSs and blue rectangles denote UTRs, respectively, whereas gray connecting lines correspond to intronic regions.

10 conserved protein motifs were detected in the 17 MdHDZIV members using MEME (Figure 4A, Table S2). Most MdHDZIV members exhibited highly similar motif compositions and arrangements, supporting the structural conservation of this family. However, differences were also observed among individual proteins. For example, *MdHDZIV11* and *MdHDZIV17* lacked specific motifs, suggesting potential functional diversification among certain members of the MdHDZIV family. Consistent with the motif variation, gene length and exon number also varied substantially among MdHDZIV members (Figure 4B). Most *MdHDZIV* genes contained multiple exons, whereas *MdHDZIV11* and *MdHDZIV17* displayed relatively simplified gene structures with fewer exon regions. In contrast, several members, including MdHDZIV3, MdHDZIV8, and MdHDZIV14, possessed more complex exon–intron structures. Taken together, the consistency among phylogenetic relationships, conserved domain organization, motif composition, gene structure, and predicted three-dimensional structure indicates that MdHDZIV proteins are structurally conserved, providing a reliable foundation for subsequent functional analyses.

### 2.3. Intraspecific Collinearity Analysis of MdHDZIVs

**Figure 5:**
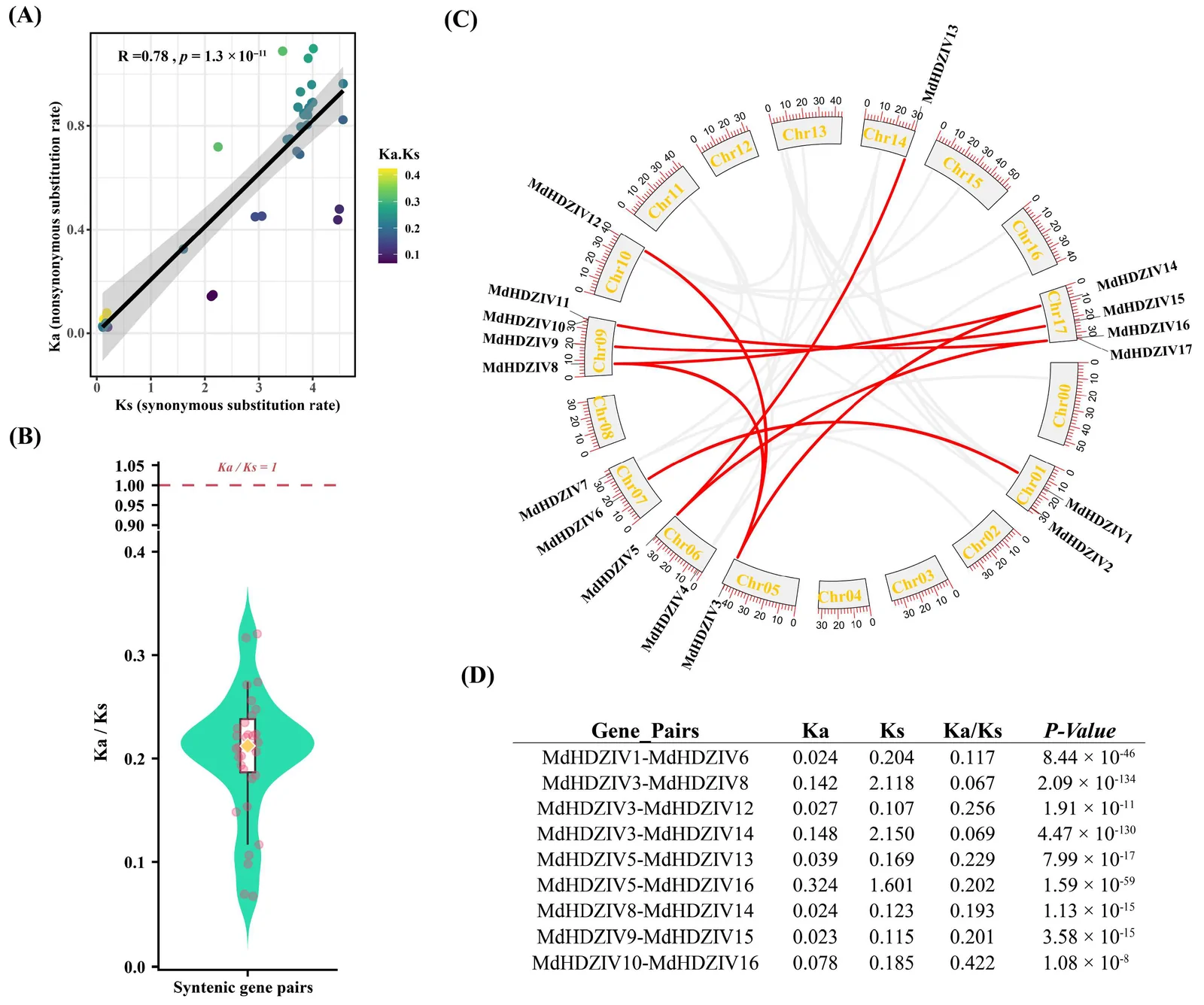
Evolutionary analysis and intraspecies collinear relationships of the MdHDZIV family in apples. (**A**) The relationship between Ka and Ks substitution rates of syntenic gene pairs containing *MdHDZIVs* in apples. (**B**) Distribution of Ka/Ks ratios for syntenic gene pairs involving MdHDZIVs in apples. The violin plot illustrates the distribution density of Ka/Ks values, while the embedded boxplot shows the median and interquartile range. The red dashed line indicates the boundary of neutral evolution, corresponding to a Ka/Ks value of 1. (**C**) The locations of 17 MdHDZIVs are indicated on the corresponding chromosomes. Red links denote segmentally duplicated gene pairs identified by collinearity analysis. (**D**) Ka and Ks substitution rates of duplicated MdHDZIV pairs.

Genome-wide syntenic relationships within the apple genome were analyzed to elucidate the evolutionary origin of the *MdHDZIV* gene family. In total, 35 homologous gene pairs associated with MdHD-ZIP IV genes were identified across the genome (Figure 5A; Table S3). The synonymous substitution rate (Ks) was significantly positively correlated with the nonsynonymous substitution rate (Ka) (R = 0.78, *p* = 1.3 × 10^−11^), suggesting that synonymous and nonsynonymous substitutions accumulated in a coordinated manner among these duplicated gene pairs during evolution. Across the homologous pairs, Ka/Ks ranged from 0.07 to 0.42, with a mean of approximately 0.21 (Figure 5B). Every duplicated MdHDZIV pair had a Ka/Ks value below 1, consistent with strong constraint under purifying selection. Collinear pairs involving *MdHDZIV* genes were then extracted from the genome-wide homology set to clarify relationships within the family (Figure 5C). Among the 17 *MdHDZIV* genes, nine collinear gene pairs were identified: *MdHDZIV1*–*MdHDZIV2*, *MdHDZIV3*–*MdHDZIV13*, *MdHDZIV5*–*MdHDZIV17*, *MdHDZIV8*–*MdHDZIV15*, *MdHDZIV8*–*MdHDZIV16*, *MdHDZIV9*–*MdHDZIV14*, *MdHDZIV10*–*MdHDZIV15*, *MdHDZIV10*–*MdHDZIV16*, and *MdHDZIV12*–*MdHDZIV13* (Figure 5D). Because these syntenic pairs spanned multiple chromosomes, segmental duplication appears to have been a major driver of expansion and diversification in the apple MdHDZIV family.

### 2.4. Analysis of Cis-Acting Elements in the Upstream Promoter Regions of Apple MdHDZIVs

**Figure 6:**
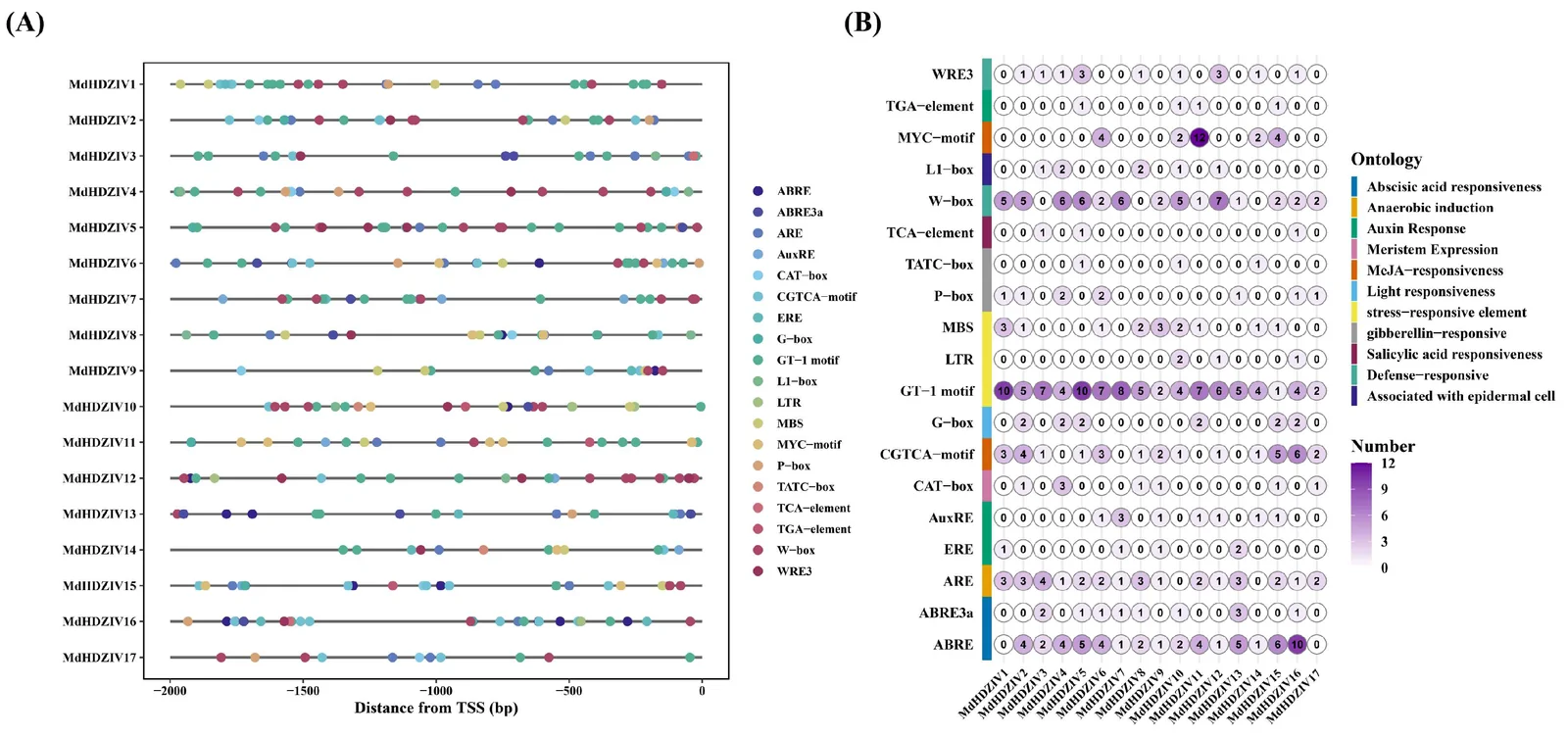
Analysis of cis-acting regulatory elements in the promoter regions of the MdHDZIV family. (**A**) Predicted cis-regulatory elements in the 2 kb upstream promoter regions of the *MdHDZIV* genes. Each circle color denotes one class of cis-acting elements. (**B**) Heatmap showing how predicted cis-regulatory elements are distributed among *MdHDZIV* promoters.

To investigate regulation of MdHDZIV transcription, the 2 kb region upstream of each family member was screened for cis-regulatory elements with PlantCARE (http://bioinformatics.psb.ugent.be/webtools/plantcare/html/ accessed on 1 January 2002) (Figure 6A; Table S4). The predicted cis-regulatory elements were classified into several functional groups associated with hormone signaling, stress responses, light regulation, and plant development, etc. Element composition and distribution differed substantially among promoters, pointing to regulatory diversification within the family. Promoters of *MdHDZIV* genes contained several stress-associated motifs, such as ABRE, ARE, GT-1, LTR, and W-box elements. Among these, ABRE and ARE elements were relatively abundant, implying potential participation of *MdHDZIV* genes in ABA-dependent regulation and responses to oxygen limitation. *MdHDZIV4*, *MdHDZIV5*, and *MdHDZIV16* were notable for relatively dense ABRE and other stress-related motifs, implying distinct regulation by stimuli such as drought, low temperature, and pathogen challenge. In addition, hormone- and light-responsive elements, including AuxRE, MYC, TCA, and G-box elements, were widely distributed across *MdHDZIV* promoters, implying that these genes may be integrated into regulatory networks associated with auxin, jasmonic acid, salicylic acid, and light signaling. The promoter regions also contained L1-box motifs in several *MdHDZIV* genes, including *MdHDZIV3*, *MdHDZIV4*, *MdHDZIV8*, *MdHDZIV10*, and *MdHDZIV12*. As L1-box elements are closely associated with epidermis-specific gene expression and epidermal cell differentiation, their presence suggests that these MdHDZIV members may participate in conserved regulatory pathways related to epidermal development, including cuticle formation and epidermal cell fate determination (Figure 6B). Collectively, these findings indicate that *MdHDZIV* genes may be regulated by both conserved epidermal development-related cis-elements, particularly L1-box elements, and diverse hormone- and stress-responsive regulatory modules, highlighting their potential roles in coordinating epidermal differentiation and environmental adaptation in apple.

### 2.5. Tissue-Specific Expression Patterns of Apple MdHDZIVs

**Figure 7:**
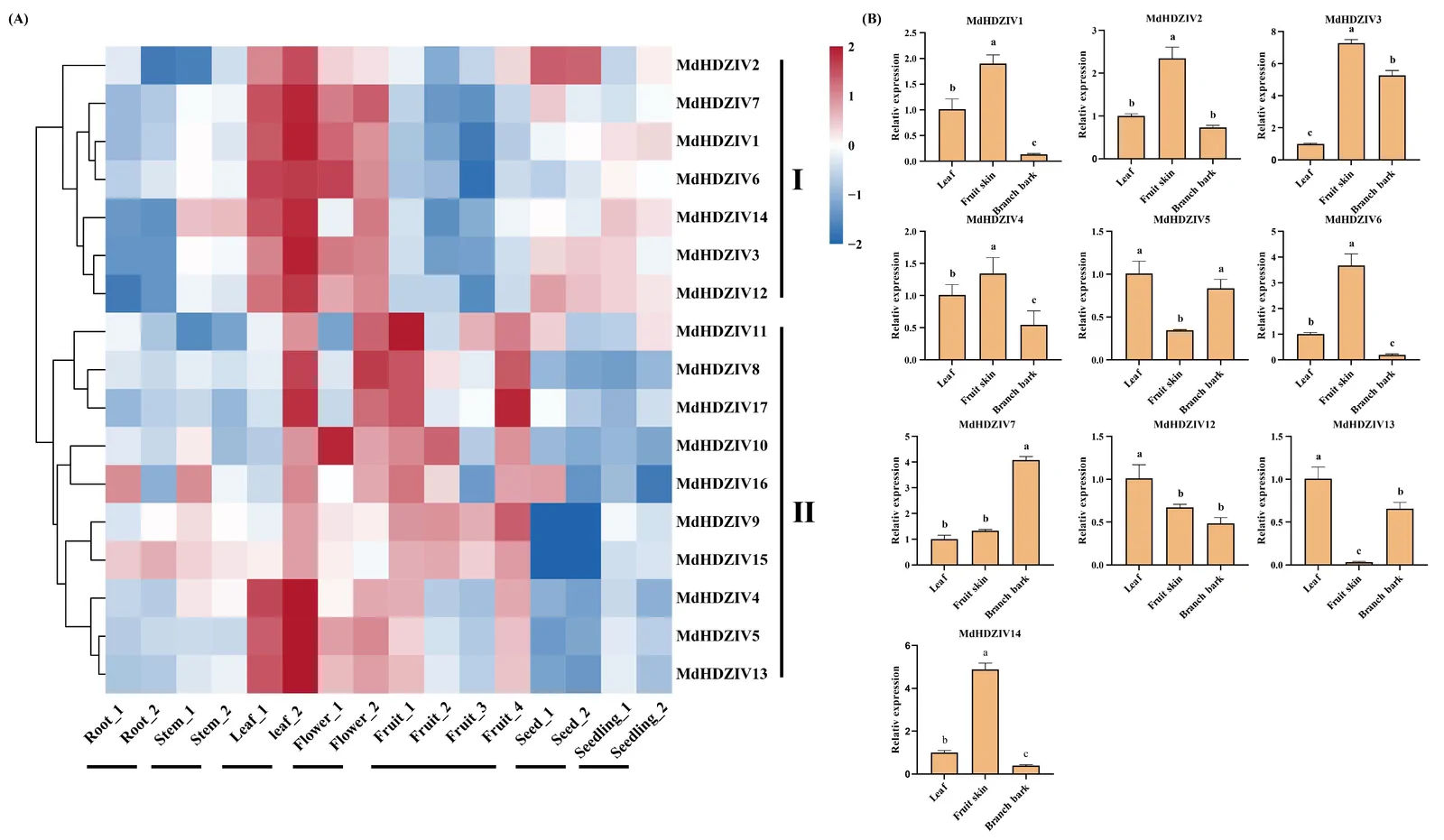
Tissue-specific expression profiles of the *MdHDZIVs*. (**A**) The heatmap presents MdHDZIV transcript abundance across apple tissues using RNA-seq data from GSE42873. Genes were hierarchically clustered according to their expression profiles and classified into two major groups (Groups I and II). Expression values were normalized as Z-scores. (**B**) qRT-PCR measurement of representative leaf-enriched *MdHDZIV* genes in leaf, fruit skin, and branch bark. Data are presented as the mean ± SD (*n* = 3). Different letters indicate significant differences (*p* < 0.05).

To define organ-specific expression of the MdHDZIV family, RNA-seq data for different apple tissues were obtained from the GEO dataset GSE42873 (Table S5). Candidate *MdHDZIV* genes showing relatively high expression in leaves were further selected for qRT-PCR validation in leaf, fruit skin, and branch bark. The clustered heatmap revealed clear expression divergence among *MdHDZIV* genes across tissues and separated the family members into two major expression groups: Group I and II (Figure 7A). Group I genes generally exhibited higher transcript levels in leaf and several developmental tissues, whereas their expression was relatively low or specifically reduced in fruit-related tissues and branch bark. In contrast, Group II genes showed relatively low or stable basal expression across tissues, suggesting that these members may be associated with more general or maintenance-related functions. Cuticular waxes are essential components of the epidermal barrier, and leaves represent typical aerial organs with active epidermal development and wax deposition. Therefore, *MdHDZIV* genes with relatively high expression in leaves were considered potential candidates involved in epidermis-related processes. Based on this rationale, 10 leaf-enriched *MdHDZIV* genes were chosen for qRT-PCR comparison among leaf, fruit skin, and branch bark (Figure 7B). The selected genes displayed clearly different expression preferences among the three tissues. *MdHDZIV3* showed the most pronounced expression differences between leaf and the other two epidermis-related tissues, fruit skin and branch bark, whereas *MdHDZIV13* displayed significantly lower expression in fruit skin than in leaf. Overall, some *MdHDZIV* genes maintained relatively high transcript levels in both leaf and fruit skin, whereas others showed preferential expression in leaf or branch bark. These tissue-dependent expression patterns suggest potential functional divergence among MdHDZIV members in different epidermal barrier-related tissues of apple.

### 2.6. Expression Patterns of MdHDZIV Genes Under Abiotic Stress

**Figure 8:**
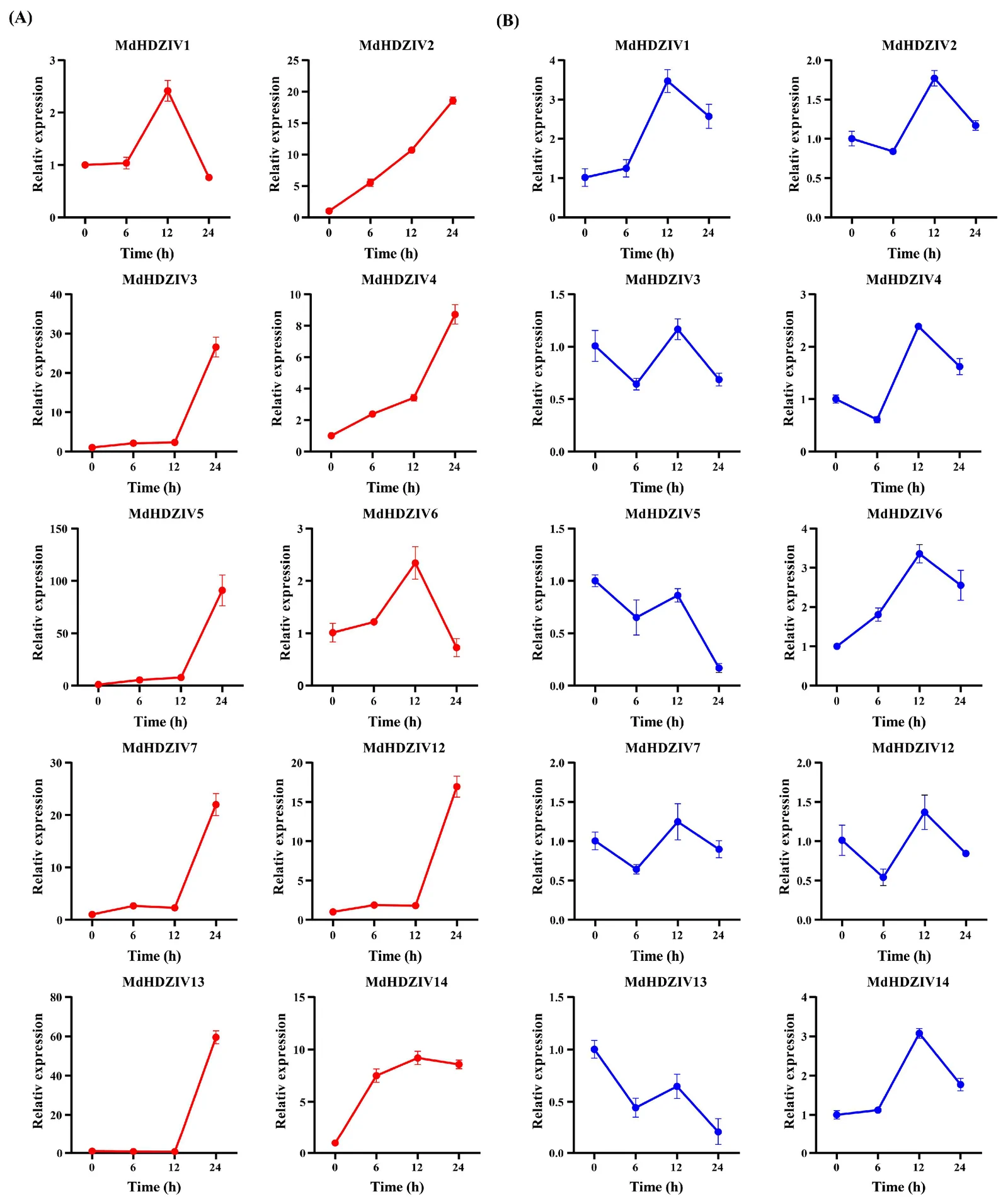
Temporal expression profiles of selected *MdHDZIV* genes in apple leaf under abiotic stress-related treatment. Relative expression levels of ten representative *MdHDZIV* genes in apple leaf over a 24 h time course (0, 6, 12, and 24 h) under 15% PEG_6000_-induced osmotic stress (**A**) and 200 mM NaCl-induced salt stress (**B**). Data are presented as the mean ± SD (*n* = 3).

Abiotic-stress responses of 10 candidate *MdHDZIV* genes were quantified by qRT-PCR at 0, 6, 12, and 24 h following PEG_6000_ and NaCl treatment. The results revealed distinct transcriptional responses among *MdHDZIV* genes under the two treatments (Figure 8). Overall, PEG_6000_ treatment elicited stronger transcriptional induction than NaCl treatment, with most responsive genes showing marked upregulation at later time points. Under PEG_6000_ treatment, *MdHDZIV2*, *MdHDZIV3*, *MdHDZIV4*, *MdHDZIV5*, *MdHDZIV7*, *MdHDZIV12*, *MdHDZIV13*, and *MdHDZIV14* were upregulated to varying degrees. Among them, *MdHDZIV3*, *MdHDZIV5*, and *MdHDZIV13* showed the greatest induction, especially after 24 h, implicating them in adaptation to osmotic stress. In contrast, *MdHDZIV1* and *MdHDZIV6* showed transient induction, with transcript levels peaking at 12 h and declining markedly thereafter (Figure 8A). Under NaCl treatment, *MdHDZIV1*, *MdHDZIV2*, *MdHDZIV4*, *MdHDZIV6*, and *MdHDZIV14* displayed moderate upregulation around 12 h, whereas *MdHDZIV3*, *MdHDZIV5*, and *MdHDZIV13* showed weak responses or decreasing expression trends (Figure 8B). These findings suggest that *MdHDZIV* genes exhibit stress type-specific and gene-specific expression patterns, indicating functional divergence among apple HD-Zip IV family members in abiotic stress responses.

### 2.7. MdHDZIV3 Enhances Tolerance to PEG-Induced Osmotic Stress

**Figure 9:**
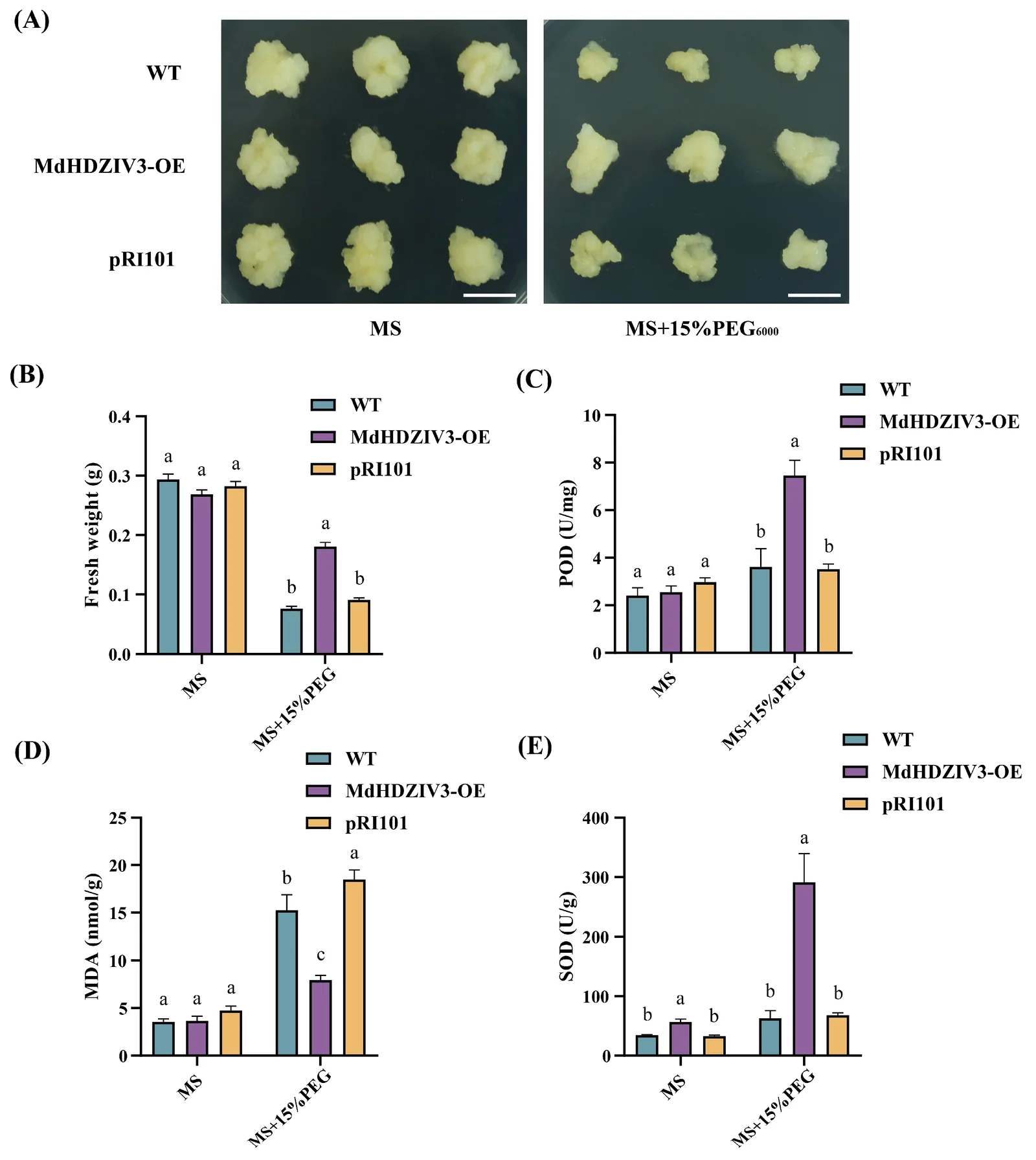
Functional characterization of *MdHDZIV3* in apple callus under PEG-induced osmotic stress. (**A**) WT, *MdHDZIV3*-OE, and empty vector controls (pRI101) grown under normal MS medium and MS supplemented with 15% PEG_6000_. Scale bars = 1 cm. (**B**–**E**) Physiological and biochemical analyses of callus under control and PEG treatment conditions, including fresh weight (**B**), peroxidase (POD) activity (**C**), malondialdehyde (MDA) content (**D**), and superoxide dismutase (SOD) activity (**E**). Data are presented as the mean ± SD (*n* = 3). Different letters indicate significant differences (*p* < 0.05).

Based on the stress-responsive expression profiles, *MdHDZIV3*, which was strongly induced by PEG_6000_ treatment, was selected for functional validation. Under normal MS medium conditions, WT, *MdHDZIV3*-OE, and empty-vector pRI101 calli showed no significant differences in morphology or fresh weight. However, under MS medium supplemented with 15% PEG_6000_, *MdHDZIV3*-OE calli exhibited enhanced growth performance, with larger callus size and significantly higher fresh weight than WT and empty-vector controls (Figure 9A,B). Physiological analyses further showed that PEG_6000_ treatment significantly increased POD (Figure 9C) and SOD (Figure 9D) activities in *MdHDZIV3*-OE calli, whereas MDA (Figure 9E) accumulation was markedly reduced compared with the control materials. The results indicate that overexpression of *MdHDZIV3* enhances antioxidant capacity and alleviates membrane lipid peroxidation under osmotic stress, thereby improving PEG tolerance in apple calli. Together with the PEG-induced expression pattern, the functional validation results indicate that *MdHDZIV3* may act as a positive regulator involved in the osmotic stress response in apple.

## 3. Discussion

### 3.1. Structural Conservation and Evolution of the Apple HD-Zip IV Gene Family

HD-Zip IV TFs represent a distinct subfamily of the plant-specific HD-Zip family. Studies across Arabidopsis, maize, tobacco, banana, cannabis, and patchouli indicate that HD-Zip IV proteins typically possess HD, LZ, START, and SAD domains but do not contain the MEKHLA domain that is characteristic of HD-Zip III members. These structural features are closely associated with their conserved roles in epidermal development, cuticle formation, lipid metabolism, and environmental adaptation [21,33,34,35,36,37]. Functional characterization of individual family members has further demonstrated their involvement in epidermal specification and environmental-stress regulation. In *Arabidopsis*, *GLABRA2* controls trichome formation and root-hair patterning, while *ATML1* and *PDF2* participate in epidermal identity establishment and formation of the embryonic epidermis [38,39]. Constitutive activation of *AtHDG11/EDT1* also increases drought resistance through modification of root-system architecture and a reduction in stomatal density [40]. These findings suggest that HD-Zip IV genes function as key regulators linking epidermal development with environmental adaptation. We therefore conducted a genome-scale investigation to clarify the composition and possible functions of the apple HD-Zip IV family.

We identified 17 *MdHDZIV* genes in apple, a number comparable to the family sizes reported for Arabidopsis, maize, and several other species [21,37]. All encoded proteins displayed the characteristic structural features of HD-Zip IV members. In phylogenetic analysis, the apple HD-Zip IV proteins clustered with Arabidopsis and rice homologues, demonstrating conservation in sequence organization and evolutionary affinity. Such conservation is consistent with the established roles of HD-Zip IV TFs in epidermal development and environmental adaptation in land plants. The predicted three-dimensional structure of *MdHDZIV3* further supported the conserved modular architecture of this family, with the HD-LZ module likely mediating DNA binding and protein dimerization, whereas the START/SAD region may contribute to lipid-related signal perception or conformational stability [15,41]. Evolutionarily, both ancient genome duplication and local duplication appear to have contributed to enlargement of the apple HD-Zip IV family. The apple genome has undergone whole-genome duplication events, which have provided an important genetic basis for the expansion and functional diversification of transcription factor families [42,43]. In the present study, both tandem and segmental duplication events were detected among MdHDZIV members, suggesting that the expansion of this family resulted from the combined effects of post-duplication gene retention, segmental duplication, and local tandem duplication rather than from a single evolutionary mechanism. During long-term evolution, duplicated genes may undergo functional redundancy, subfunctionalization, or neofunctionalization [44,45]. The uniformly low Ka/Ks ratios of duplicated MdHDZIV pairs suggest strong purifying selection and, consequently, retention of their central protein functions. Therefore, functional divergence among MdHDZIV members is more likely to have arisen from variation in promoter regulation, tissue-specific expression, and stress-responsive expression patterns than from rapid changes in protein-coding sequences.

### 3.2. Cis-Regulatory Features Suggest Functional Diversification of MdHDZIV Genes

Promoter cis-acting element analysis provided additional evidence for the potential regulatory diversification of *MdHDZIV* genes. The occurrence of ABRE, ARE, GT-1, LTR, W-box, MYC, G-box, and related motifs indicates that MdHDZIV transcription may be connected with hormone-signaling and environmental-stress networks. Tobacco and patchouli display similar enrichment of hormone- and stress-related promoter motifs, and several HD-Zip IV genes in these species alter their transcript abundance after stress or exogenous-hormone treatment [35,36]. Notably, L1-box elements were detected in the promoters of *MdHDZIV3*, *MdHDZIV4*, *MdHDZIV8*, *MdHDZIV10*, and *MdHDZIV12*. Given that the L1-box is a key cis-acting element associated with epidermis-related gene expression [35,37,39], these five genes may contribute to epidermal cell specification or establishment of the apple surface barrier.

### 3.3. Organ-Specific Expression Indicates Potential Roles in Epidermal Barrier Formation

Expression profiling is a valuable approach for inferring functional divergence among members of a gene family. Many HD-Zip IV genes are enriched in epidermal or subepidermal cells and participate in trichome differentiation, root-hair patterning, cuticle assembly, wax production, and organ-specific surface protection [15,16,21,36]. In our dataset, the differential expression of *MdHDZIV* genes across apple organs suggests that these members may have undergone functional specialization during tissue development and environmental adaptation. Leaf, fruit skin, and branch bark are directly exposed to external environments and therefore require well-developed epidermal barriers and wax deposition for surface protection. Accordingly, *MdHDZIV* genes highly expressed in these tissues may be closely associated with epidermal barrier formation, wax metabolism, and stress adaptation. Moreover, the distinct expression preferences of MdHDZIV members among leaf, fruit skin, and branch bark indicate potential subfunctionalization or tissue-specific regulatory divergence within this family. These expression patterns provide a useful basis for identifying candidate *MdHDZIV* genes involved in epidermal development, wax deposition, and environmental responses in apple.

### 3.4. MdHDZIV Genes Exhibit Distinct Responses to Osmotic and Salt Stress

Abiotic stress expression profiling further supported functional divergence among MdHDZIV members. Under PEG_6000_-induced osmotic stress and NaCl-induced salt stress, the 10 candidate *MdHDZIV* genes displayed distinct transcriptional response patterns. Overall, PEG_6000_ treatment elicited stronger and more sustained induction than NaCl treatment. In particular, *MdHDZIV3*, *MdHDZIV5*, and *MdHDZIV13* were markedly upregulated at 24 h under PEG_6000_ treatment, whereas *MdHDZIV1* and *MdHDZIV6* exhibited transient induction, with expression peaking at 12 h and declining thereafter. By contrast, NaCl treatment induced relatively moderate expression changes, with most responsive genes showing increased transcript levels around 12 h. These divergent responses may reflect the different physiological effects of the two stress treatments: PEG_6000_ primarily imposes water deficit and osmotic stress, whereas NaCl stress involves both osmotic imbalance and ion toxicity. The stronger transcriptional response to PEG_6000_ suggests that some MdHDZIV members may be preferentially involved in dehydration- or ABA-associated osmotic stress signaling. Similar stress-responsive divergence has been reported for HD-Zip IV genes in banana and tobacco, where different family members respond distinctly to drought, salt, temperature, and hormone treatments [33,36]. Together, these findings indicate that *MdHDZIV* genes exhibit gene-specific and stress type-specific expression patterns, suggesting functional specialization within this family during abiotic stress responses.

### 3.5. MdHDZIV3 May Positively Contribute to PEG-Induced Osmotic Stress Tolerance

Among the stress-responsive MdHDZIV members, *MdHDZIV3* was selected for preliminary functional validation because of its relatively high expression in epidermis-related organs and strong induction under PEG_6000_ treatment. Under normal MS medium conditions, WT, empty-vector, and *MdHDZIV3*-OE calli showed no apparent differences in morphology or fresh weight. However, under MS medium supplemented with 15% PEG_6000_, *MdHDZIV3*-OE calli exhibited improved growth performance and significantly higher fresh weight than the control materials. Physiological analyses further showed that *MdHDZIV3* overexpression significantly increased POD and SOD activities while reducing MDA accumulation under PEG_6000_-induced osmotic stress. Because SOD and POD participate in reactive-oxygen detoxification and MDA reflects lipid-peroxidation damage, the observed changes indicate that *MdHDZIV3* may improve osmotic tolerance by reinforcing antioxidant defense and protecting cellular membranes. The involvement of the related *Arabidopsis* genes *ATML1* and *PDF2* in epidermal and wax regulation raises the possibility that *MdHDZIV3* may also contribute to stress adaptation through pathways associated with epidermal barrier formation or wax metabolism. However, the regulatory targets and molecular mechanism of *MdHDZIV3* remain to be further elucidated.

### 3.6. Overall Implications and Future Perspectives

At the genome-wide level, we systematically defined the composition, structural organization, evolutionary relationships, and expression-regulatory patterns of the apple HD-Zip IV subfamily. The MdHDZIV proteins displayed typical structural features of HD-Zip IV members and showed evidence of strong purifying selection, indicating that this family is evolutionarily conserved in apple. Combined promoter and expression pattern evidence links *MdHDZIV* genes with epidermal processes, wax-associated metabolism, and responses to abiotic stress. Among these genes, *MdHDZIV3* was strongly induced by PEG_6000_ treatment, and its overexpression in apple calli increased fresh weight, enhanced antioxidant enzyme activities, and reduced MDA accumulation under PEG_6000_-induced osmotic stress, suggesting a potential positive role in osmotic stress tolerance. While the callus system successfully confirms the gene’s general stress defense mechanisms, future studies on organized aerial tissues (such as leaves or fruit peel) will help fully clarify its direct role in structural wax and cuticle dynamics. Overall, this work establishes a systematic framework for understanding the apple HD-Zip IV subfamily and offers candidate gene resources for future studies of epidermal development, wax regulation, and environmental adaptation.

## 4. Materials and Methods

### 4.1. Identification of Apple HD-Zip IV Family Members

Protein sequences for *Malus domestica* (‘Golden Delicious’, GDDH13_protein_v1.1) were downloaded from the Genome Database for Rosaceae (GDR). The ‘Golden Delicious’ GDDH13 reference proteome was selected because of its high-quality genome assembly and comprehensive gene annotation, which provide a reliable genomic resource for genome-wide gene family identification in apple. Pfam provided the HMM profiles for the conserved HD and START domains; individual profiles were extracted with ‘hmmfetch’ under Linux. The apple proteome was screened against the HMM profiles using the ‘hmmsearch’ program implemented in HMMER [46], with an E-value cutoff of 1 × 10^−5^. All retrieved proteins were subsequently examined using the NCBI CD-Search tool to confirm the presence of the conserved HD and START domains. After domain confirmation, 17 HD-Zip IV genes were identified and renamed according to their physical positions. Chromosomal distribution maps were generated using the MG2C software (version 2.1) [47].

### 4.2. Analysis of Physicochemical Properties of MdHDZIV Proteins

Physicochemical attributes of the MdHD-Zip IV proteins were characterized using the pepstats program (v6.6.0) and the EMBOSS package under a Linux environment with default parameters [48]. MW, Protein length, pI, and other physicochemical parameters were calculated automatically. Subcellular localization was predicted using CELLO_v2.5 [49].

### 4.3. Phylogenetic Analysis

HDZIV protein sequences from *Arabidopsis thaliana* and rice (*Oryza sativa*) were obtained from TAIR and the China Rice Data Center, respectively. These sequences, together with the MdHDZIV protein sequences, were aligned using Muscle (v3.1). MEGA X was used to infer an ML phylogeny under the JTT+G+F model with 1000 bootstrap replicates; all other parameters remained at default values. The final tree was subsequently displayed and annotated using the iTOL v.7 online platform [50].

### 4.4. Gene Structure and Conserved Motif Analysis

MEME Suite v5.5.6 was used to identify conserved motifs in MdHD-Zip IV proteins [51]. The search was limited to 10 motifs, allowed motif widths of 6–50 amino acids, and used the ‘any number of repetitions’ occurrence setting; all other options were unchanged. Gene structure information, including exon, intron, and UTR organization, was obtained from the apple genome annotation file (GDDH13_v1.1). The distributions of conserved motifs together with gene structural features were integrated and displayed using TBtools II (version 2.096) [52].

### 4.5. Collinearity and Gene Duplication Analysis

Genome-wide collinearity analysis was conducted using MCScanX v1.0.0. [53] to examine gene duplication patterns and evolutionary relationships of the apple HD-ZIP IV family. The GDDH13 genome annotation and corresponding protein sequence files were provided as input for the identification of duplicated gene pairs. Segmental duplication events were inferred based on collinear blocks detected by MCScanX. To evaluate selection pressure acting on duplicated HD-Zip472 IV gene pairs, CDSs were aligned according to their corresponding protein sequence alignments. The Ka, Ks, and Ka/Ks ratio were calculated using KaKs_Calculator_v1.2 [54] with the Yang–Nielsen (YN) model.

### 4.6. Analysis of Upstream Promoter Regions

Promoter sequences spanning 2 kb upstream of the transcription start sites (TSSs) of the *MdHDZIV* genes were retrieved using SeqKit_2.8.0 [55]. These sequences were subsequently analyzed with the PlantCARE database [56] to predict cis-regulatory elements. The distribution of cis-acting elements was subsequently visualized using R v4.3.0 scripts.

### 4.7. RNA Extraction and qRT-PCR

For tissue-specific expression analysis, leaf, fruit skin, and branch bark samples were collected from the hybrid progeny of ‘Jonathan’ × ‘Golden Delicious’ at the Beidaihe Experimental Station of China Agricultural University. Progeny with smooth, non-russeted fruit skin and an intact epidermal structure were selected because these materials provide a suitable biological background for evaluating the epidermis-associated expression patterns of MdHDZIV genes. For abiotic stress treatments, two-month-old soil-grown GL-3 seedlings were subjected to three treatment conditions: untreated control, 15% PEG_6000_, and 200 mM NaCl. Following the treatments, samples were harvested at four time points (0, 6, 12, and 24 h). Total RNA isolation was carried out with the Universal Plant Total RNA Kit (EasyPure). Subsequently, 2 μg of purified RNA was used as the template for first-strand cDNA synthesis with PrimeScript™ RT Master Mix (Perfect Real Time; TaKaRa, Kusatsu, Japan). qRT-PCR was performed using TB Green Premix DimerEraser (TaKaRa), with *MdActin3* (MD12G1140800) used as the internal reference gene. Relative expression levels were calculated using the 2^−ΔΔCt^ method. The primers are listed in Table S6.

### 4.8. PEG-Induced Osmotic Stress of Calli

WT, empty-vector pRI101, and *MdHDZIV3*-OE transgenic calli were transferred onto standard MS medium or MS medium supplemented with 15% PEG6000 to evaluate their responses to PEG-induced osmotic stress. One independent MdHDZIV3-overexpressing callus line was used in this experiment. Replicate samples were derived from the same transgenic callus line. The calli were cultured under dark conditions in a tissue culture room for approximately 15 days. After treatment, callus growth performance was recorded, and samples were collected for subsequent physiological analyses.

### 4.9. Determination of Callus Fresh Weight, MDA Content, and SOD and POD Activities

Fresh weight, MDA content, SOD activity, and POD activity were measured in WT, empty-vector pRI101, and *MdHDZIV3*-OE calli after treatment. Callus fresh weight was measured using an analytical balance with 0.0001 g precision. MDA content, SOD activity, and POD activity were determined using an MDA content assay kit (BC6415), an SOD activity assay kit (BC0175), and a POD activity assay kit (BC0090), respectively.

### 4.10. Statistical Analysis

All measurements were performed with three biological replicates. Statistical analyses were conducted using SAS 9.4, and graphs were generated using GraphPad Prism 8.0.

## Figures and Tables

**Table 1:** Characteristics of *MdHDZIV* genes in apples.

Gene	ID	Start	End	Exon Number	Amino Acid	Mw (KDa)	pI	GRAVY	Subcellular Location
*MdHDZIV1*	MD01G1050200	15310065	15316370	9	823	89.32	6.22	−0.314	Nuclear: 2.615
*MdHDZIV2*	MD01G1198100	29598802	29604966	9	766	84.39	5.28	−0.250	Cytoplasmic: 1.920 Nuclear: 1.403
*MdHDZIV3*	MD05G1342200	46260424	46265963	11	736	80.85	5.75	−0.316	Nuclear: 3.245
*MdHDZIV4*	MD06G1025900	3125763	3131658	12	834	92.82	6.01	−0.386	Nuclear: 4.049
*MdHDZIV5*	MD06G1236400	36780228	36784227	10	710	78.43	6.59	−0.322	Nuclear: 3.406
*MdHDZIV6*	MD07G1127600	18026791	18033492	9	824	89.56	6.40	−0.314	Nuclear: 2.566
*MdHDZIV7*	MD07G1266200	33439790	33444768	9	808	87.74	5.57	−0.195	Cytoplasmic: 1.425 Nuclear: 1.300
*MdHDZIV8*	MD09G1107300	7816050	7821927	13	749	81.63	5.98	−0.344	Nuclear: 2.235
*MdHDZIV9*	MD09G1209300	20171374	20175770	11	762	84.64	6.19	−0.497	Nuclear: 3.770
*MdHDZIV10*	MD09G1277500	35416146	35425520	11	734	81.65	6.45	−0.329	Nuclear: 2.914
*MdHDZIV11*	MD09G1277600	35429844	35432916	9	659	74.65	7.34	−0.208	PlasmaMembrane: 1.724 Nuclear: 1.430
*MdHDZIV12*	MD10G1315800	39934035	39938635	12	740	80.93	6.03	−0.296	Nuclear: 3.005
*MdHDZIV13*	MD14G1243300	32184593	32188605	10	709	78.36	6.88	−0.235	Nuclear: 3.081
*MdHDZIV14*	MD17G1095000	8045867	8051741	14	756	82.59	5.19	−0.360	Nuclear: 2.360
*MdHDZIV15*	MD17G1191000	22799324	22803876	11	761	84.54	5.46	−0.494	Nuclear: 3.693
*MdHDZIV16*	MD17G1271000	33174787	33178890	11	696	77.48	7.37	−0.274	Nuclear: 1.981
*MdHDZIV17*	MD17G1271100	33180914	33184248	9	635	71.66	6.63	−0.329	Nuclear: 3.131

## Data Availability

The original contributions presented in this study are included in the article. Further inquiries can be directed to the corresponding authors.

## Supplementary Materials

The following supporting information can be downloaded at: https://www.mdpi.com/article/10.3390/plants15172670/s1, Table S1: Identification of HB and START domains in MdHD-Zip IV family proteins; Table S2: Table of Motif distribution in MdHDZIVs; Table S3: Genome-wide detection of collinear MdHDZIVs pairs; Table S4: Cis-acting elements table about 2 kb promoter regions of MdHDZIVs; Table S5: Tissue-specific expression patterns based on transcriptome data from the GEO database; Table S6: Table of markers used to qRT-PCR primers.
